# Simulation-Based Education as a Solution to Challenges Encountered with Clinical Teaching in Nursing and Midwifery Education in Malawi: A Qualitative Study

**DOI:** 10.1155/2024/1776533

**Published:** 2024-06-24

**Authors:** Gertrude Mwalabu, Annie Msosa, Ingrid Tjoflåt, Kristin Hjorthaug Urstad, Bodil Bø, Eva Christina Furskog-Risa, Patrick Mapulanga, Masauko Msiska

**Affiliations:** ^1^Department of Adult Health Nursing, School of Nursing, Kamuzu University of Health Sciences, Lilongwe, Malawi; ^2^Department of Quality and Health Technology, Faculty of Health Sciences, University of Stavanger, Stavanger, Norway; ^3^Department of Public Health, Faculty of Health Sciences, University of Stavanger, Stavanger, Norway; ^4^Department of Caring and Ethic, Faculty of Health Sciences, University of Stavanger, Stavanger, Norway; ^5^Library Department, Kamuzu University of Health Sciences, Lilongwe, Malawi; ^6^Department of Biomedical Sciences, School of Life Sciences and Allied Health Professions, Kamuzu University of Health Sciences, Lilongwe, Malawi

## Abstract

Nursing and midwifery education in Malawi entails theoretical learning and clinical practice, essential for developing competent professionals. However, challenges such as staff shortages and limited resources hinder effective clinical teaching. Simulation-based education (SBE) offers a promising solution. This study aims to explore how SBE can enhance clinical teaching in Malawian nursing and midwifery education. Data were collected through in-depth interviews with lecturers, clinical instructors, and focus group discussion (FGD) with the final-year students. Thematic analysis revealed several key findings: SBE serves as a valuable gap-filler in clinical education, addressing complex needs while offering diverse learning opportunities. It also provides a platform for enhanced supervision and assessment strategies. The results indicate that SBE enables students to master various clinical skills without direct patient contact, reducing congestion at clinical sites while ensuring credit acquisition. Moreover, it proves effective as both a supervision and assessment tool for evaluating students' clinical performance. In conclusion, the study advocates for the integration of SBE into Malawian nursing and midwifery education to alleviate the challenges associated with traditional clinical teaching. By leveraging SBE, institutions can mitigate overcrowding at clinical sites and provide students with diverse learning experiences. However, successful implementation requires adequate infrastructure, resources, and skilled lecturers. Ultimately, SBE holds the potential to significantly enhance the quality and outcomes of nursing and midwifery education in Malawi.

## 1. Introduction

Nursing and midwifery are complex professions that require knowledge, affective attitudes, and psychomotor skills for safe professional practices and optimal patient outcomes [1]. The training of nurses and midwives in Malawi is organised in such a way that students are taught theoretical modules before they are exposed to aspects in clinical settings [2]. Students can choose to do a program that combines nursing and midwifery, or they can train as midwives or nurses separately. The transfer of theoretical knowledge into practice is a crucial part of preparing students for nursing and midwifery practice. This process is facilitated by clinical placement in various health facilities where students undergo clinical teaching and supervision [3]. The clinical aspects of nursing and midwifery training enable student nurses and midwives to grow professionally by allowing them to put their theoretical knowledge into practice, thus applying theory to real-world situations [4]. The literature recommends that clinical instructors in health facilities teach or guide students during their clinical placement to achieve the necessary competencies [5].

During clinical placements, nurse/midwifery lecturers and clinical instructors are expected to collaborate to facilitate students' clinical learning experiences and development of clinical skills. According to the Nurses and Midwives Council of Malawi clinical guidelines, ten students are supposed to be supervised by one clinical instructor during clinical placement. However, with a large student population coupled with staff shortages, limited clinical sites, and material resources, it is not possible to effectively provide quality clinical support, as stipulated by the professional regulatory body. This compromises the quality and quantity of clinical teaching in Malawi [4]. Therefore, the clinical support and teaching of students by both academic and clinical staff is reported to be unsatisfactory [4].

Bø et al. [6] indicate that SBE is useful in the acquisition of nursing and midwifery skills in resource-limited countries. Other scholars have described SBE as an evidence-based pedagogical tool that is effective in the acquisition of clinical skills and thoroughly prepares students to train in a safe environment that is not hazardous to patient safety [7].

The Nurses and Midwives Council of Malawi developed SBE guidelines to improve the acquisition of skills by students in training institutions and health facilities. However, apart from the guidelines, there is limited literature on innovative teaching strategies in Malawi that can mitigate the challenges encountered in clinical teaching. Therefore, this study was conducted to explore how SBE can improve clinical teaching in nursing and midwifery education in Malawi.

## 2. Methodology

### 2.1. Design

This study used an exploratory descriptive qualitative design to explore how SBE can improve clinical teaching in nursing and midwifery education in Malawi. According to [8], an exploratory descriptive qualitative design is used when not much has been done in the area under study. In the current study, the design was considered appropriate because there is a paucity of literature on the use of SBE in relation to the clinical teaching of nurses and midwives in Malawi.

### 2.2. Study Population

The study population was comprised of the following:

- Fourth-year nursing and midwifery students
- Lecturers in nursing and midwifery training institutions
- Clinical instructors in health facilities

### 2.3. Inclusion Criteria

The inclusion criteria include the following:

- Nursing and midwifery students in the final year of their program and aged 18 years and above
- Clinical instructors and lecturers with more than six months of work experience in selected hospitals and training institutions, respectively

### 2.4. Exclusion Criteria

The exclusion criteria include the following:

- Nursing and midwifery students aged below 18 years in years 2–4 of their programs
- Clinical instructors and lecturers with less than six months of work experience in selected hospitals and training institutions, respectively

### 2.5. Sampling Strategy

Purposive sampling was used to identify lecturers, clinical instructors, and final-year nursing and midwifery students who participated in this study. Purposive sampling is a method in which the researcher deliberately selects participants based on specific criteria [9]. Clinical instructors were approached by nursing officers responsible for different health facilities to meet the research team and provide consent for their participation in the study. Lecturers and students were recruited through the heads of the department and the office of the students' dean, respectively.

### 2.6. Data Collection

Data were collected through in-depth interviews and focus group discussion (FGD) which were audio-recorded with permission from the participants. FGD and in-depth interviews were conducted at the participants' institutions by appointment. The main focus of the FGD and in-depth interviews were the participants' knowledge about SBE problems being experienced in clinical teaching and how SBE can help to deal with some of the problems. Each FGD and in-depth interview lasted approximately 60 min and 45 min, respectively. Thirty-five in-depth interviews were conducted with 24 lecturers and 11 clinical instructors, using the same semistructured interview guide. A total of 90 final-year nursing and midwifery students participated in ten FGDs, with each group having between 8 and 10 participants (Table 1). All participants in the FGD thoroughly discussed each issue before moving to another issue to give all participants an opportunity to make their contributions.

This study was part of a larger study which focused on challenges encountered in clinical teaching in Malawi and gaps in the nursing and midwifery curricula in Malawi that could be filled using simulation. However, the focus of the current study was to explore feasible solutions to mitigate the challenges in clinical teaching and provide suggestions on how simulation could improve clinical teaching in Malawi.

### 2.7. Data Management

All audiotaped data were stored in soft copies on multiple computers before transcription to prevent data loss. The audiotaped raw data were then transcribed verbatim to prepare the information for analysis. Member checking was performed on ten conveniently selected study participants after transcription. This was done in order for the participants to verify the information that was collected from them.

### 2.8. Data Analysis

Data analysis was based on Clarke and Braun's [10] thematic analysis framework which has six steps. It was conducted by the principal investigator (PI) and a research assistant, who was a nurse midwife with vast experience in qualitative research methods. The PI and research assistant independently analysed the data from in-depth interviews and FGD. They began by familiarising themselves with the data by reading the transcripts to obtain a clear understanding of the data. In the process, the two identified words and phrases which appeared frequently in the transcripts and coded them as they were deemed to represent specific ideas. Coded words and phrases were later scrutinised and grouped according to their commonalities. Each group was given a heading which was considered as a tentative theme. The tentative themes were reviewed by two researchers, the principal investigator and research assistant, to identify the final themes, as indicated in Table 2.

### 2.9. Ethical Considerations

Ethical approval for this study was obtained from the College of Medicine Research and Ethics Committee (P.07/21/3362). In addition to ethical approval, permission to access the participants was obtained from responsible officials in the participating health facilities and training institutions. Verbal and written informed consent were obtained from each participant before the interview. To maintain the privacy of the participants, data were collected in designated institutional rooms. The researchers also ensured that the names of participants were not written in any research document and were not associated with any information that was collected from the respective participants. Participants had the right to withdraw from the study without any negative consequences.

## 3. Results

### 3.1. Participant Demographic Characteristics

Results show that 52 (57.7%) of the participants who took part in FGD were aged between 21 and 25 years, and 76.6% (69) were female. On the other hand, 40% (14) of the participants in the in-depth interviews were aged between 36 and 40 years, and 65.7% (23) of them were female (Table 2). In terms of qualifications, 48% (17) were holders of a bachelor of science, while in terms of work experience, 34.3% (12) of the participants had between 11 and 14 years of work experience and with some kind of previous experience with SBE at the time of the interviews.

### 3.2. Themes

Following a comprehensive analysis, which involved amalgamating data from individual interviews with those from FGD to establish unified descriptors, four primary themes and their corresponding subthemes emerged, as delineated in Table 3 and explained below. These themes and subthemes were elucidated through quotations extracted from FGD and in-depth interviews. Table 3 lists the themes that emerged from these data.

### 3.3. SBE as a “Gap-Filler”

Participants indicated that SBE can help fill the gaps in clinical teaching. This theme emerged from the four subthemes listed in Table 2. Lecturers and clinical instructors explicitly elaborated that with SBE, an overloaded curriculum would no longer be a challenge. They indicated that using scenarios and objectives in an SBE provides an opportunity to effectively cover several topics in one scenario. In addition, participants indicated that hospitals and other clinical sites would be relieved of the burden of high student numbers, as some of the activities would be performed at simulation sites.

“Simulation could be ideal in lightening the pressure of our overloaded curricula because we can cover so many topics with just one scenario. The good thing is that so many students can learn so many topics through one scenario; even if they are withdrawn from the clinical sites to work in the simulation centres or skills laboratory to ease the clinical congestion, the hours would still be counted by Nurses and Midwives Council of Malawi.” Senior Lecturer 5.

Clinical instructors indicated that working in teams with real patients is not always possible in a real clinical environment, where some trainee health professionals may decide to work independently of others. Therefore, they felt that SBE would help students from different professions and training institutions work in teams.

“The more students simulate, the more they develop multi-disciplinary skills, how to communicate and relate with other professionals, which is currently missing in our nursing and midwifery education. Simulation provides a more practical—hands-on experience of more skills in one scenario involving other professionals such as medical officers, dietitians, physiotherapists, radiologists even laboratory staff which is not possible with other teaching methods.” Clinical instructor 3.

Clinical instructors and lecturers acknowledge that there are knowledge and skill gaps, particularly for some clinical emergencies, conditions, and skills in clinical settings. This was confirmed by qualified nurses and midwives, who felt that SBE can significantly help expose students to conditions and emergencies that are rare in the clinical setting.

“Some qualified nurses/midwives have gone into practice without experiencing some conditions/skills in the college or clinical settings. Like some of us, we spent our entire college life without coming across procedures/skills like managing shoulder dystocia. With simulation, nurses and midwives can have a chance to practice and get oriented to these rare conditions or skills in teams or with a friend (mentorship) in the skills labs or simulation rooms.” Clinical instructor 6.

Several clinical instructors have noted that simulated patient-based education could potentially mitigate the language barrier they confront while engaging in teaching sessions with students or healthcare professionals from non-English-speaking countries. The role-playing that is done during simulation makes it easier for those who are not proficient in English to understand what is happening through action.

“There are times when international students or health professionals from other French-speaking countries like Burundi, Congo come for clinical orientation in our health facilities through NMCM (the regulatory body) before being licensed to practice in Malawi; they do not fluently speak English, use of simulation could reduce language barrier if it is only reinforced…” Clinical instructor 3.

### 3.4. Needs in Clinical Teaching

The participants, mostly students, felt that there were some needs in clinical teaching which could be addressed by SBE. They highlighted the need to adopt innovative teaching strategies, lecturers, and clinical instructors to properly master nursing and midwifery skills. They suggested the adoption of innovative teaching strategies to improve students' acquisition of skills during clinical placement. They believed that SBE was an innovative strategy that could effectively assist students in integrating theory and practice.

“Clinical teaching remains a challenge in Malawi as it demands masterly of skills apart from having knowledge. Most lecturers as they grow professionally, they shun away from bedside care to office work because they may be deficient in some nursing and midwifery skills depending on how they were taught the skills; so they are good at knowledge not skills but with simulation that challenge can be reduced because it exposes nursing and midwifery students to a lot of practical activities which have the potential to greatly improve their nursing and midwifery skills.” Student 13.

Most students felt that frequent use of SBE would help lecturers and clinical instructors be fully equipped with theoretical knowledge that would help them properly guide students during SBE sessions. It would also help them master nursing and midwifery skills which could be an advantage for the students.

“They say practice makes things perfect and I think this can also be applied to teaching clinical skills by our lecturers. The more they teach using simulation, the more skills and knowledge they gain because they will be doing it now and again. So, I feel that in addition to us, as students gain the required skills using simulation, it is also time for our teachers to further sharpen their clinical skills and knowledge” Student 31.

### 3.5. Opportunities for SBE

Both lecturers and students felt that, during theory sessions in training institutions, students did not have adequate opportunities to practice in skills laboratories because of limited time and space. Therefore, participants suggested the establishment of SBE centres that could be accessible to students at any time in all training institutions.

“In the case of our institution, we have over 1,500 students who would want to practice some skills in the skills laboratory which can only hold about 20 students at a time… makes it somehow difficult for all the students to use the laboratory. The time required for laboratory testing is also limited. If I was given an opportunity to advise management, I would say we need a purposefully built simulation centre which is well equipped and where students can practice skills at any time they want.” Lecturer 7.

According to the study participants, most health facilities do not have rooms where skills can be practiced before students start practising with real patients. The participants indicated that such rooms would help gauge students' skills before they started dealing with patients. Therefore, they suggested that health facilities should provide special rooms for skill acquisition through SBE.

“During training, time for skills labs is not adequate… Simulation corners in health facilities can significantly help in skill acquisition. I have provided evidence through the two training sessions I attended on the SBE. Students in South Africa and Tanzania benefit from simulation corners situated within health facilities. Our students lack simulation corners for skills acquisition after theory block….it is a matter of liaising with hospital management to create space for skills acquisition within health facilities.” Senior Lecturer 2.

### 3.6. Strengthened Supervision and Assessment Strategy

This study found that SBE can be used as a strategy for the supervision and assessment of students' clinical learning. The participants indicated that simulation can provide students with an opportunity to rehearse skills and recognise their weaknesses and strengths through feedback from colleagues. They further indicated that in all these, the clinical instructor or lecturer would be there to supervise a clinical activity and provide assistance where needed.

“With simulation, lecturers can supervise and assess our competencies concurrently as they provide feedback, while we evaluate ourselves on how we have performed to perfect our skills, current teaching methods miss on this.” Student 9.

This study found that SBE can be used to assess students' knowledge and skills. The participants reported that a simulation session can give the clinical instructor or lecturer an idea about the students' level of knowledge and skills, depending on their performance in a simulation session. Such an assessment would help make proper decisions about students to attain the expected clinical learning outcomes.

“Simulation is very effective in assessing and evaluating knowledge and skill acquisition. If students grasp the desired outcomes of a particular topic or skill, they will act in accordance with the given instructions and demonstrate their acquired knowledge or skills. In this way, lecturers can assess students. Students will also be able to self-assess their learning” Clinical instructor 1.

## 4. Discussion

The aim of this study was to explore how SBE can be used to improve clinical teaching in Malawi and to discuss the major findings of the study in relation to the available literature.

### 4.1. SBE and Gaps in Clinical Teaching in Malawi

The findings indicate that SBE helps fill the gaps in clinical teaching in Malawi. According to this study, such gaps are related to overloaded curricula, overcrowded clinical sites, working in teams, clinical teaching of rare skills and conditions, and language barriers. Pivač et al. [11] highlight that the use of scenarios in SBE helps teachers to cover more clinical skills and topics in a given time as opposed to the traditional ways of teaching skills. Similarly, a quasi-experimental study conducted in Tanzania asserted that a well-facilitated simulation provides learners with a wide range of high-quality experiences through a single scenario and promotes critical thinking, reasoning, and decision-making while also strengthening hands-on skills [12]. In addition, inadequate clinical sites, such as hospitals, make them overcrowded with students from various training institutions. Jacob et al. [13] conducted a study in Tanzania to gauge the perceptions of students about their clinical environment where it was found that among others, students had negative perceptions about their clinical environment because of congestion. Similar findings were reported in South Africa by Fadana and Vember [14], who indicated that overcrowding reduces students' opportunities to practice with patients. On the other hand, Mbakaya et al. [15] argued that student overcrowding at clinical sites limits their acquisition of skills and competencies. In such situations, SBE would be an alternative to relieve such clinical sites of the pressure of having too many students.

In modern times, working in multidisciplinary or interprofessional teams is advocated [16, 17]. It is believed that simulations which involve trainees from different professions help students learn from one another, understand the roles of other professions, and perfect their skills in patient care [18]. Multidisciplinary teams would also help students improve their communication skills, interpersonal relationships, and leadership skills [19]. These skills can be transferred to the workplace, thereby enhancing patient care. Therefore, the use of SBE would help to bridge the interprofessional gap in the Malawian clinical setting where teamwork of students from different disciplines is lacking, according to the current study.

This study has shown that it is not always possible for students in a clinical setting to find certain clinical conditions on which they can practice [20]. In addition, a lack of knowledge and skills regarding uncommon conditions among students and healthcare workers could be a recipe for poor medical care [21]. Therefore, SBE fills the gap by providing students with scenarios which can mimic rare or uncommon conditions, situations, or unusual complications in the clinical setting. Such scenarios require students to make proper diagnoses and provide the right healthcare plan. Sanges et al. [22] developed an innovative approach which was successful in teaching rare diseases to medical students using role-play simulations.

The study findings have shown that SBE can fill a gap related to language and culture in clinical teaching in Malawi. While clinical teaching may focus on students' acquisition of critical skills and competencies related to their profession, it is very easy to ignore some skills which on the surface may not seem to be crucial on the surface. Nurses, midwives, and other healthcare providers may come into contact with clients from diverse language and cultural backgrounds which can present a problem in the provision of care due to the inability to understand clients [23]. As such, students in healthcare professions are supposed to be equipped with proper skills that would help them deal with clients who may have a different language and cultural background from their own. Therefore, SBE can be used to train healthcare students on how they can provide quality healthcare to culturally diverse groups of clients or clients with whom they do not share a common language [24].

### 4.2. Mastery of Both Knowledge and Skills

The study participants felt that the use of innovative teaching strategies, such as simulation, in nursing and midwifery education can help improve clinical teaching. This is in line with research conducted on obstetric neonatal emergency simulations in rural India by Zhong et al. [25], who recommended the use of simulation in the training of healthcare workers. Simulation is an effective pedagogical approach that provides students with hands-on linkages between theory and practice [26], and thus helps them develop competency in nursing and midwifery. Studies have highlighted the value of simulations in the training of healthcare workers [27–29]. In a study that evaluated a simulation-based education program with final-year undergraduate nursing and midwifery students Plummer et al. [30] reported that the use of SBE improves students' decision-making skills, critical thinking skills, motivation, confidence, and experience in real-life practice. Similarly, a meta-synthesis about the appropriateness of SBE in the training of radiologists in South Africa, by Hazell et al. [31] found that simulation increased students' confidence.

The findings of this study highlight the significance of lecturers' and clinical instructors' mastery of knowledge and skills in improving clinical teaching. For clinical teaching to achieve the intended outcomes, lecturers and clinical instructors should have the appropriate knowledge and skills which should be imparted to students. Hill [32] attests that role-playing in an obstetric emergency can be challenging for both lecturers and students, because of the complexity and urgency of the situation. Such challenging situations require professionals who are knowledgeable and skilled. Several studies have reported that SBE is a good strategy for teaching and fostering the development and acquisition of knowledge and skills [6, 33, 34]. This implies that formal SBE training for all lecturers and clinical instructors in all training institutions and settings should be emphasised.

### 4.3. SBE Infrastructure

Both lecturers and students in the current study felt that, during theory, students did not have adequate time to practice skills in training institutions, and even during clinical allocation, the students did not adequately assist in sharpening their skills and improving their nursing and midwifery competencies before they started assisting patients. Most health facilities and training institutions do not have infrastructure in which skills can be practiced before and during clinical placements. These findings resonate with Toale et al. [35], who further opine that one way in which SBE can facilitate clinical teaching is through the establishment of simulation corners within health facilities which can provide students with ample time to master their clinical skills. Studies have shown that SBE can be conducted in actual clinical settings in designated rooms to provide students with the opportunity to practice clinical skills and increase their proficiency in both technical and nontechnical skills [36, 37].

### 4.4. SBE as a Supervision and Clinical Assessment Strategy

The study participants alluded to the fact that SBE is a good strategy for supervising students' clinical activities and assessing how well they are performing in the activities. The purpose of clinical supervision in the training of nurses, midwives, and other health professionals is to help students develop as professionals in their respective training fields [38]. According to Bifarin and Stonehouse [39], clinical supervision is a process in which students who are doing clinical work are properly supported by teachers and other clinical instructors to attain and improve the skills necessary for their profession. The use of SBE in student supervision helps students gain a deeper understanding of issues and concepts and improves their problem-solving and decision-making skills through scaffolding [20].

Supervision and assessment in education go hand in hand, although their aims differ [40]. While supervision mostly focuses on supporting, coaching, and facilitating student learning, assessment is mainly concerned with making judgments about student learning and achievement of certain milestones or objectives. Ryall et al. [41] conducted a systematic review to examine the use of simulation for assessment of technical skills in health professional education where it was found that simulation-based clinical assessments can be used to evaluate students' performance and skills acquisition. In addition, Koster and Soffler [42] highlighted that because of other assessment barriers in the real clinical environment, simulations are becoming a valuable means for assessing students' competences and skills. In the case of Malawi, one of the barriers that could reduce students' opportunities to be assessed in an authentic clinical situation, as highlighted elsewhere, could be the congestion of students at clinical sites. In a study conducted in Canada to investigate simulation utilisation and evaluation practices and approaches among undergraduate nursing educational programs, Zitzelsberger et al. [43] indicated that participants reported that simulation was a valuable strategy for the evaluation of students' learning and performance. Therefore, with careful planning, clinical assessment using simulations in Malawi can become a reliable way of making judgments about students' clinical learning.

In conclusion, SBE is a possible solution to some of the challenges encountered in clinical teaching in Malawi. Despite the many challenges posed by clinical teaching, SBE is consistently believed to mitigate these challenges and add value to clinical teaching through improvements in knowledge and skill acquisition. This study suggests that simulation centres and corners should be established in all training institutions and health facilities to provide lecturers and students with ample time to bolster their clinical skills, thereby improving the quality of patient care.

### 4.5. Study Limitations

This study was mostly conducted in training institutions and health facilities where researchers constantly interacted with the study participants. To some extent, some of the collected data may have been influenced by this prior interaction. However, the involvement of other training institutions not affiliated with researchers' institutions has remedied this limitation. Furthermore, some data collectors had no prior interaction with the participants. Second, this study presents the perspectives of the lecturers, clinical instructors, and students. This was a limitation in terms of adequately and properly amalgamating the themes from all three groups of participants. However, analysis of the data by experienced people ensured that the analysis was performed with great care to capture views from all groups of participants.

## Figures and Tables

**Table 1:** Proportion of study participants in relation to the population for each participant group.

Participant group	Total population	Sampled participants	Percentage
Students	639	90	14
Clinical instructors in health facilities	702	11	1.5
Lecturers	136	24	18
Total	1477	125	8

**Table 2:** Participant demographic characteristics.

Age	Number of participants	Relative frequency (%)
*Participants for FGDs*		
18 years to 20 years	0	0
21 years to 25 years	52	57.7
26 years to 30 years	25	27.7
Above 30 years	13	14.3
Total	90	100
Gender		
Male	21	23.3
Female	69	76.6
Total	90	100

*Participants for in-depth interviews*		
Age		
25 years to 30 years	1	2.9
31 years to 35 years	3	8.6
36 years to 40 years	14	40.0
41 years to 45 years	10	28.6
46 years to 50 years	6	17.0
Above 50 years	1	2.9
Total	35	100
Gender		
Male	12	34.3
Female	23	65.7
Total	35	100
Qualifications		
BSc level	17	48.0
MSc level	14	40.0
PhD level	4	11.4
Total	35	100
Work experience		
6 months to 5 years	7	20.0
6 years to 10 years	8	22.9
11 years to 14 years	12	34.3
15 years to 19 years	6	17.1
Above 20 years	2	5.7
Total	35	100

**Table 3:** Themes and subthemes on how SBE can be used to improve clinical teaching in Malawi.

Themes	Subthemes
SBE—a gap-filler	(1) Lightening the pressure of an overloaded curriculum through the use of scenarios (2) Students working in teams using a multidisciplinary approach (3) Training rare clinical skills (4) Mitigating language barriers in teaching international students

Needs in clinical teaching	(1) Innovative teaching strategies (2) Mastery of nursing and midwifery skills for lecturers and clinical instructors

Opportunities for SBE	(1) Establishment of simulation corners in health facilities (2) Establishment of simulation centres in training institutions

Strengthened supervision and assessment strategy	(1) SBE for clinical supervision (2) SBE for clinical assessment

## Data Availability

The data used to support the findings of this study are included within the article.
